# Nutritional correlates of the overwintering and seaward migratory decisions and long-term survival of post-spawning Atlantic salmon

**DOI:** 10.1093/conphys/coz107

**Published:** 2019-12-18

**Authors:** X Bordeleau, B G Hatcher, S Denny, F G Whoriskey, D A Patterson, G T Crossin

**Affiliations:** 1 Department of Biology, Dalhousie University, 1355 Oxford Street, P.O. Box 15000, Halifax, NS B3H 4R2, Canada; 2 Bras d'Or Institute for Ecosystem Research, Cape Breton University, 1250 Grand Lake Road, Sydney, NS B1M 1A2, Canada; 3 Unama'ki Institute of Natural Resources, 4102 Shore Rd, Eskasoni, NS B1W 1C2, Canada; 4 Ocean Tracking Network, Dalhousie University, 1355 Oxford Street, P.O. Box 15000, Halifax, NS B3H 4R2, Canada; 5 Fisheries and Oceans Canada, Cooperative Research Management Institute, Resource and Environmental Management, Simon Fraser University, Burnaby, BC V5A 1S6, Canada

**Keywords:** Acoustic telemetry, carryover effects, life history trade-offs, plasma triglycerides, previously spawned

## Abstract

Despite the importance of iteroparity (i.e. repeated spawning) for the viability of Atlantic salmon populations, little is known about the factors influencing the migratory behaviour and survival prospect of post-spawned individuals (kelts). To test the hypothesis that post-spawning nutritional condition underlies differences in spatiotemporal aspects of the habitat use and survival of migrating Atlantic salmon kelts, we physiologically sampled and acoustically tagged 25 individuals from the Middle River, Nova Scotia in autumn 2015. Kelts were subsequently tracked within their natal river during the winter months, and as far as 650 km away along known migration pathways towards the Labrador Sea and Greenland. Some kelts were detected nearly 2 years later, upon their return to the natal river for repeat spawning. Overall, kelts in poor or depleted post-spawning nutritional state (i.e. low body condition index or plasma triglyceride level): (i) initiated down-river migration earlier than higher condition kelts; (ii) experienced higher overwinter mortality in the natal river; (iii) tended to spend greater time in the estuary before moving to sea and (iv) did not progress as far in the marine environment, with a reduced probability of future, repeat spawning. Our findings suggest that initial differences in post-spawning condition are carried through subsequent migratory stages, which can ultimately affect repeat-spawning potential. These results point to the importance of lipid storage and mobilisation in Atlantic salmon kelts for mediating post-spawning migratory behaviour and survival.

## Teaser

Lay summaries We tagged and physiologically sampled 25 Atlantic salmon kelts to test the hypothesis that differences in post-spawning condition are carried through their subsequent migration. We found that kelts in poor nutritional condition migrated down-river sooner and died to a greater extent in freshwater and at sea, ultimately affecting repeat-spawning potential.

## Introduction

A predominant life history trade-off involves the differential investment of energy reserves into current reproduction versus those required for self-maintenance, survival and/or future reproductive potential (Stearns, 1989). In sexually reproducing organisms, this trade-off is particularly important for capital breeders, which rely on somatic energy reserves accrued prior to breeding to power migrations to breeding areas, produce gametes and support the behaviour necessary for successful courtship and mating (Stearns, 1989; Jager *et al.,* 2008).

Whilst high-reproductive investment likely increases the probability of current reproductive success, it can exact costs in terms of post-breeding mortality and a reduced likelihood of future reproduction (i.e. costs of reproduction; Williams, 1996). At one end of the spectrum, the ultimate investment into a single life-time reproductive event (i.e. semelparity) is favoured where there is low variability in juvenile survival and low post-breeding survival. However, in other circumstances there can be benefits to conserving resources to increase the probability of breeding more than once (i.e. iteroparity) (Cole, 1954; Murphy, 1968). As such, with increasing variability in the survival of offspring, natural selection favours life histories that spread the risk of reproductive failure over space or time (Murphy, 1968; Stearns, 1976).

This life history trade-off between current reproduction and survival is well illustrated within the semelparity—iteroparity continuum of salmonid fishes (family *Salmonidae*), where high total energy expenditure during reproduction is negatively associated with the probability of future (repeat) breeding which is lowest in Atlantic salmon (*Salmo salar*) and steelhead trout (*Oncorhynchus mykiss*) at 10–11% and highest in brown trout (*Salmo trutta*) and Arctic char (*Salvelinus alpinus*) at 34–41%, amongst iteroparous species (Fleming, 1998; Fleming and Reynolds, 2004). This continuum can also manifest within populations of the same species, as illustrated in Atlantic salmon where the average total energy expenditure for reproduction was negatively correlated with the average post-spawning survival rate of a population (Jonsson *et al.,* 1997). Whilst inter-population differences in the post-spawning survival of salmonids can result from differences in habitat (Jonsson *et al.,* 1991) and/or anthropogenic disturbances (Maynard *et al.,* 2017, Bordeleau *et al.*, 2019), these are at least partly caused by variation in life-history traits (e.g. sea-age at first maturity) and body size that influences reproductive investment, which then affect post-spawning survival and population demographics (Fleming and Reynolds, 2004; Jonsson and Jonsson, 2011a). As such, smaller early-maturing salmon (i.e. one sea winter, or 1SW) that invest proportionally less into reproduction (40–60% of total energy reserve) than larger multi sea winter individuals (up to 70% of total energy reserve) generally show higher post-spawning survival (Jonsson *et al.,* 1991, 1997; Fleming, 1998; Jonsson and Jonsson, 2003, 2011a). This is further corroborated by a recent study that identified a genotypic co-inheritance between sea-age at maturity and iteroparity in Atlantic salmon, with iteroparity being more likely in smaller, earlier-maturing salmon that invest proportionally less into reproduction (Aykanat *et al.,* 2019).

For iteroparous salmonids, post-spawning energetic condition is believed to be an important mediator of migratory decision making and subsequent survival (Belding, 1934; Jonsson *et al.,* 1997; Halttunen *et al.,* 2013; Bordeleau *et al.,* 2018a). After spawning in the fall, energetically depleted Atlantic salmon kelts spend variable amounts of time in freshwater before initiating their seaward migration. Some may initiate seaward migration soon after spawning, whilst others might overwinter in fresh water and delay migration until the spring (Jonsson and Jonsson, 2011b). Although overwintering in fresh water is a common strategy for kelts, the winter months are generally lean with few feeding opportunities. Kelts must therefore rely primarily on somatic lipid reserves to sustain basic metabolic costs (Jonsson *et al.,* 1997), pointing towards the importance of post-spawning energetic condition in mediating migratory decisions and survival. As such, Halttunen *et al.* (2013) reported that Atlantic salmon kelts with low body condition exited freshwater soon after spawning in an attempt to restore their depleted state via estuarine or marine foraging, whilst kelts in better condition delayed migration until the spring and opted for overwintering in freshwater. Once they have left freshwater, residency and habitat use in estuaries and near shore marine areas can also be highly variable amongst and within anadromous salmonid populations. For example, inter-individual variation in the estuarine residency of Atlantic salmon kelts can range from a few days (Hubley *et al.,* 2008) to upwards of several weeks (Hedger *et al.,* 2009), and even months (Bordeleau *et al.,* 2018b), with high inter-individual variability.

Environmental cues (e.g. river discharge, water temperature and photoperiod) can correlate with the timing of seawater entry of juvenile salmonid smolts, which tends to occur each year within a narrow window (e.g. few weeks) (Jensen *et al.,* 2012; Otero *et al.,* 2014). However for kelts, the temporal window for migration is wide (e.g. autumn and spring migrants) and less predictable and does not appear to be linked to discreet environmental cues, suggesting an increased importance of endogenous factors in mediating post-spawning migratory decisions. When subjected to nutritional constraints, a major source of metabolic energy comes from the production of glycerol and free fatty acids via the hydrolysis of plasma triglycerides, which were released into circulation from the catabolism of lipids stored in adipose and muscle tissue (Sargent *et al.,* 2002). During starvation, the concentration of triglycerides in tissues and in circulation diminishes due to the energy demand imposed by basal metabolic processes (Kakizawa *et al.,* 1995). As such, plasma triglyceride concentration has been shown to be a useful, non-lethal indicator of nutritional status in many taxa, including wild salmonids (Boel *et al.,* 2014; Gauthey *et al.,* 2015; Bordeleau *et al.,* 2018a). Recently, triglyceride levels have been linked to inter-individual variation in the marine habitat use of post-spawned anadromous brown trout with depleted individuals remaining at sea for longer periods, in a presumed effort to offset higher nutritional needs (Bordeleau *et al.,* 2018a).

Whilst the timing of downriver migration to the marine environment, and to some extent the overwinter survival of Atlantic salmon kelts, have both been linked to nutritional condition and stress state (Belding, 1934; Halttunen *et al.,* 2013; Bordeleau *et al.,* 2018a; Birnie-Gauvin *et al.,* 2019), the few previous telemetry studies documenting natural variation in post-spawning condition and its influence on the migratory behaviour and survival of post-spawned Atlantic salmon were limited in time and space (i.e. to ocean entry; Halttunen *et al.,* 2013; Birnie-Gauvin *et al.,* 2019). As such, it remains unclear how post-spawning condition and the initial decision about when to initiate downstream migration then affect the marine migratory behaviour and longer-term survival of Atlantic salmon kelts that either recondition at sea for from a few months (i.e. consecutive repeat spawners), or to more than a year (i.e. alternate repeat spawners).

In Atlantic salmon, and iteroparous salmonids more broadly, repeat spawners that are mostly constituted by large females, have an important ecological role contributing disproportionally to total annual egg deposition. For example in Atlantic salmon, 3.2–13.6% repeat spawners in annual returns were estimated to contribute to 7.1–28.1% of all eggs, an influence that was accentuated in years of low maiden spawner returns (Bordeleau *et al.*, 2019). Combined with recent findings that iteroparity has been increasing in mid-latitudinal and northern parts of the Atlantic salmon’s North American range, where repeat spawners have increased from 3.1 (1971–92) to 7.6% (1993–2017) of annual returns (Bordeleau *et al.*, 2019), identifying the factors influencing the habitat use and repeat-spawning potential of Atlantic salmon would benefit current and future conservation efforts in the face of declining populations. To address current knowledge gaps, we combined acoustic telemetry and physiological sampling to test the hypothesis that post-spawning nutritional condition underlies individual differences in migratory behaviour and survival. As such, we physiologically sampled and acoustically tracked post-spawned Atlantic salmon kelts through their marine migrations (up to 650 km from the River mouth and over up to 654 days) until their eventual return as repeat spawners, which was made possible through the use of the extensive network of acoustic receiver arrays put in place by the Ocean Tracking Network and partners (Iverson *et al.,* 2019). More specifically, we tested whether the post-spawning level of the nutritional depletion of individuals (i.e. lower body condition factor or plasma triglyceride concentration) was related to the: (i) earlier initiation of downstream migration; (ii) higher overwinter mortality; (iii) extended estuarine residency and (iv) reduced probabilities of marine progression and repeat spawning.

## Methods

### Study system and acoustic receiver array

**Figure 1:**
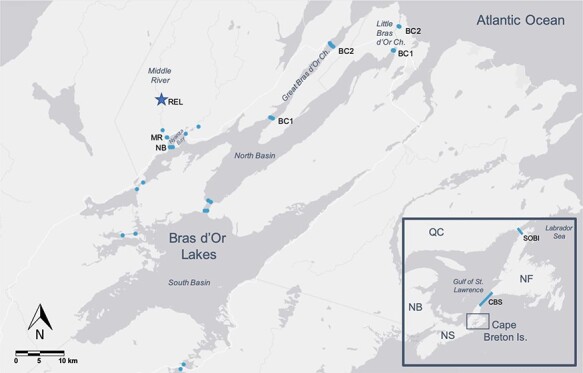
Map of the acoustic array positioned in the Bras d’Or Lakes, Nova Scotia and in the Atlantic Ocean. Acoustic receivers are indicated by blue circles in the Bras d’Or Lakes (*n* = 30) or blue lines in the Ocean, and the star symbol indicates the release location of kelts (the map is produced using Esri, HERE, Garmin, NGA, USGS). REL is release site, MR is Middle River mouth, NB is Nyanza Bay, BC1 is the first gate of either Bras d’Or channels, BC2 is the second gate of either Bras d’Or channels, CBS is Cabot Strait and SOBI is Strait of Belle Isle.

We conducted our study on the Atlantic salmon population of the Middle River, Nova Scotia, draining into the vast estuarine habitat formed by the brackish Bras d’Or Lake system (~1100 km^2^ in area, 45°51′37″N, 60°46′44″W; Fig. 1). Population numbers in this and surrounding rivers have been decreasing in recent decades, and whilst the Middle River supports the largest spawning runs remaining in the Eastern Cape Breton populations, annual returns are low (<350 on average: Gibson and Levy, 2014). The declines prompted the Committee on the Status of Endangered Wildlife In Canada (COSEWIC, 2010) to recommend that the Eastern Cape Breton Atlantic salmon population segment be designated as endangered by the Canadian government.

For seaward migrating Atlantic salmon leaving the Middle River and migrating through the Bras d’Or Lakes system, the closest access to the Atlantic Ocean is located ~64 km away, either through the Great or Little Bras d’Or Channels (Fig. 1). In order to document the general overwinter habitat use and seaward migrations of acoustically tagged Atlantic salmon kelts, a total of 30 VR2W acoustic receivers (Vemco Ltd, NS, Canada) were deployed throughout the Bras d’Or Lakes, forming acoustic detection gates (i.e. consisting in multiple receiver units crossing a waterbody) that fish must cross en route to the Atlantic Ocean (Fig. 1; see Crossin *et al.,* 2016 for additional details on the array). Once in the Atlantic Ocean, kelts could then be detected on two additional marine gates positioned across the Cabot Strait (i.e. to detect entry in the Gulf of St. Lawrence) and across the Strait of Belle Isle (i.e. the Northern exit of the Gulf of St. Lawrence towards the Labrador Sea, Fig. 1).

To evaluate the detection efficiency of the acoustic array, we examined data from the five receiver gates the fish had to cross on their way to the Gulf of St Lawrence (i.e. river mouth, Nyanza Bay, two gates in either the Great or Little Bras d’Or channels, and Cabot Strait, Fig. 1), and determined that all individuals (100%) that were detected on a given gate were also detected on the previous more inland/upstream gate. Thus, the detection efficiency of the array was high (Bordeleau *et al.,* 2018b).

### Fish capture, blood collection and tagging

A total of 25 post-spawned Atlantic salmon (51.1–95.0 cm FL) were captured on the Middle River by angling between 26 November and 10 December 2015. Immediately after capture, individual salmon were placed in a padded cradle supplied with ambient river water to collect a ~2 ml blood sample via caudal venipuncture (as described in Huston, 1990). The average time from hooking to blood collection was 8:32 min (standard deviation: 6:36 min, range: 2:49–30:49 min). Blood samples were placed in an ice-water slurry before centrifugation. Following blood collection, fish were individually anesthetized, and then underwent surgical procedures (as described in Cooke *et al.,* 2011) at the site of capture for the implantation of 69 kHz V16-4H acoustic transmitter (16 mm diameter × 68 mm, 24 g in air, nominal delay of 20–70 s, estimated battery life of 1257 days; Vemco Ltd, NS, Canada). Transmitter mass relative to body mass averaged 1.0% for kelts (range: 0.4–1.9%), well within the tolerance limits for Atlantic salmon tag burden (Lacroix *et al.,* 2004). At the end of the tagging procedure, fork length and mass were recorded, scales were collected for ageing, and sex was determined morphologically. Body condition index (BCI) was calculated from the residuals of the linear regression between log_e_ (mass in g) and log_e_ (fork length in mm) (i.e. residual mass, Kaufman *et al.,* 2007). Sea age at first maturity was determined from scale readings (White and Medcof, 1968). Following sampling and tagging, kelts were placed in a recovery basin and later released at the capture site (between 9:30 and 15:30) after regaining full equilibrium and swimming capacities (~1 h after tagging). Fish were handled in conformity with guidelines established by the Canadian Committee on Animal Care, approved by Dalhousie University and Cape Breton University Animal Care Committees (protocol numbers: 14-105 and 1213-16, respectively). They were captured under the authority of a Scientific Fishing License granted by Fisheries and Ocean Canada (number: 340450).

### Blood processing and triglyceride assay

Within 3 h of blood collection, samples were centrifuged at 1163 g for 10 min and the resultant plasma was collected and flash-frozen at −80°C. Plasma triglyceride levels were assayed in duplicate using a commercially available colorimetric kit (Cayman Chemical Company, USA) and read at 530 nm with a BioTek Synergy HTX microplate reader (BioTek Instruments, Inc., USA), according to the manufacturer’s standard procedure. The mean coefficient of variation between duplicates was 6.7% (coefficient of variation threshold of <15%).

### Data analyses

To test the hypothesis that variation in the post-spawning nutritional state of individuals was related to differences in migratory behaviour and survival, different types of models were computed (i.e. binomial logistic regression, general linear regression or Cox proportional hazards regression) depending on the nature of the response variable or aspect of interest. For each model, indices of nutritional state (i.e. plasma triglycerides level, body condition) were considered as potential explanatory variables whilst fork length, sea-age at first maturity, sex, as well as simple interactions were considered as potential covariates. Due to limited sample sizes, final statistical models were limited to the inclusion of a maximum of two explanatory variables for a minimum sample size of 20 individuals, and to a single explanatory variable for sample sizes lower than 20. The best-fitting model was selected using a stepwise model building approach (based on Akaike information criterion (AIC) values and comparison with a null model; Anderson *et al.,* 2001) using the *step* function in R v .3.5.0 (R Development Core Team, 2018). Note that plasma triglyceride concentration and BCI were not correlated (*r* = 0.08, Pearson correlation coefficient).

#### Overwintering habitat choice and downstream migration timing

After being tagged in late fall, kelts spent various amount of time upstream before initiating their downstream migration to the Middle River mouth and adjacent estuary of Nyanza Bay (Fig. 1). The initiation of downstream river migration and associated overwinter habitat choice was determined from the timing of individuals’ first detection at the river mouth, which was bimodally distributed with autumn (i.e. before 31 January) and spring downstream migrants (i.e. after 1 April) (more details in Results section). All of the autumn migrating kelts were detected at the river mouth during the winter months and none of them were detected on the Nyanza Bay gate before spring, indicating that they ceased migration and overwintered in the lower section of the river or the upper estuary, as opposed to overwintering upstream for spring downstream migrants. To test our first prediction that the initiation of the downstream migration of post-spawned Atlantic salmon was related to individuals’ nutritional condition, with kelts in poorer condition migrating down earlier, binomial logistic regression models (with two possible outcomes: autumn or spring downstream migrants) were computed in R. Model selection was conducted as previously described.

#### Overwinter riverine survival

Following the observation that no kelts were detected on estuarine receivers of the Bras d’Or Lakes or on marine gates before spring (i.e. earliest detection outside the river on April 24), the overwinter riverine survival of kelts tagged in late-November/early-December was calculated from the proportion of tagged individuals that reached the River mouth conditional to being later detected on the first estuarine gate positioned in Nyanza Bay (indicating that individuals exited the Middle River). In other words, individuals that were not detected outside of the Middle River for the following years were presumed to have died in the river. To test our second prediction that the apparent overwinter riverine survival of post-spawned Atlantic salmon was related to individuals’ nutritional condition, with kelts in poorer condition less likely to survive, binomial logistic regression models were computed in R. Model selection was conducted as previously described.

#### Estuarine residency

For overwinter riverine survivors, the estuarine residency period of individuals started from the last detection at the river mouth receiver gate (estuarine entry) and ended at the first detection at an outermost receiver gate of either one of the Bras d’Or channels (estuarine exit or Ocean entry, Fig. 1). To test the hypothesis that the duration of the estuarine residency period of post-spawned Atlantic salmon was related to individuals’ nutritional condition, with kelts in poorer condition spending more time reconditioning in the estuary before initiating their migration to oceanic feeding grounds, general linear regressions were computed in R. Model selection was conducted as previously described.

#### Marine migration and overall survival

From the initial release site, kelts had to cross four acoustic receiver gates on their way to the Atlantic Ocean (i.e. Middle River mouth, Nyanza Bay and then two consecutive gates in either the Great or the Little Bras d’Or channels, Fig. 1). Once at sea, kelts could then be detected on two additional marine gates, crossing the Cabot Strait to enter the Gulf of St. Lawrence and then crossing the Strait of Belle Isle to reach the Labrador Sea (Fig. 1). However, once in the Gulf of St. Lawrence, not all Atlantic salmon kelts migrate to the Labrador sea (Chaput and Benoit, 2012; Lacroix, 2013; Strøm *et al.,* 2017), therefore the probability of detection at the Strait of Belle Isle cannot be solely interpreted as survival as it is also confounded by the migratory decision of individuals. For that reason, a lack of detection through the Strait of Belle Isle was interpreted as a shorter migratory progression rather than directly inferring mortality. Finally, because of the long-lasting battery life of tags (>4 years), potential repeat spawners could then be re-detected at the mouth of the Middle River in following years. As such, re-detection in their natal river in the following 2 years (i.e. 2016 and 2017) was the criteria used to infer overall survival (i.e. to repeat spawning). In total, kelts could be detected on six different acoustic gates and potentially back to the Middle River mouth in subsequent years. To test the hypothesis that the migratory progression and overall survival probability of kelts throughout the different stages of migration was influenced by individuals’ nutritional condition, with kelts in better condition progressing further and more likely to spawn again, Cox proportional hazards regressions were computed using the *coxph* and *Surv* function of the *survival* package in R (Therneau, 2019). This method, originally based on time-to-event analysis, was slightly modified by using a spatial variable (i.e. gate number, from 1 to 7) instead of time such that an individual that was detected at the last Bras d’Or gate (i.e. gate no 4) but not after was marked as to have survived until *t*(4) (Lennox *et al.,* 2018). By adding explanatory variables in the model, this allowed us to evaluate the influence of nutritional condition and other covariates on the observed progression or survival pattern through the different stages of migration. Model selection was conducted as previously described and the proportional hazards assumption was verified using the *cox.zph* function of the *survival* package (testing the independence between Schoenfeld residuals and time/gate) (Grambsch and Therneau, 1994). Finally, we plotted Kaplan-Meier survival curves to visualize model output using the *survfit* function of the *survival* package in R.

## Results

### Overwintering habitat choice and downstream migration timing

Amongst the 25 Atlantic salmon kelts (19 females, 6 males) that were tagged and released in the Middle River between 26 November and 10 December 2015, post-spawning downstream migratory timing was bimodally distributed. Eight individuals initiated downstream migration in late fall or early winter (termed autumn migrants), with first detections at the river mouth gate between 29 November and 21 January (median: 7 December) depending on the individual. All of these autumn downstream migrants were detected at the river mouth during the winter months (until death or moving out in the spring) and none of them were detected on estuarine or marine receivers before spring and were therefore considered to have overwintered in the lower part of the Middle River or near its mouth. Twelve individuals spent the entire winter up-river, only initiating downstream migration in the spring (termed spring migrants) as first detected at the River mouth between 23 April and 25 May (median: 7 May). A remaining five individuals were never detected at the river mouth or elsewhere in the acoustic array and were presumed to have died in the river.

**Figure 2:**
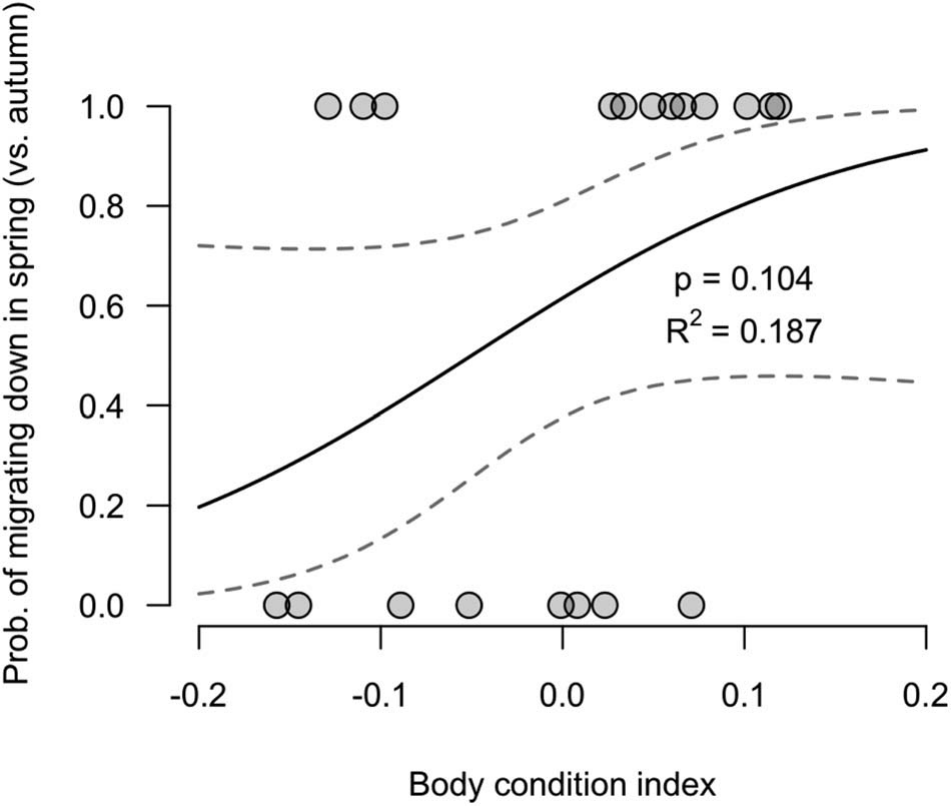
Binomial logistic regression of the probability of spring downstream migration timing vs*.* the alternative autumn migration as a function of BCI (as a numerical variable—for visualisation purposes only as it was not the best-fitting model, Table 1). The coefficient of determination (*R*^2^) of the logistic regressions was computed using the *lrm* function of the *rms* package in R (Nagelkerke, 1991). Dashed lines represent the 95% CI.

**Figure 3:**
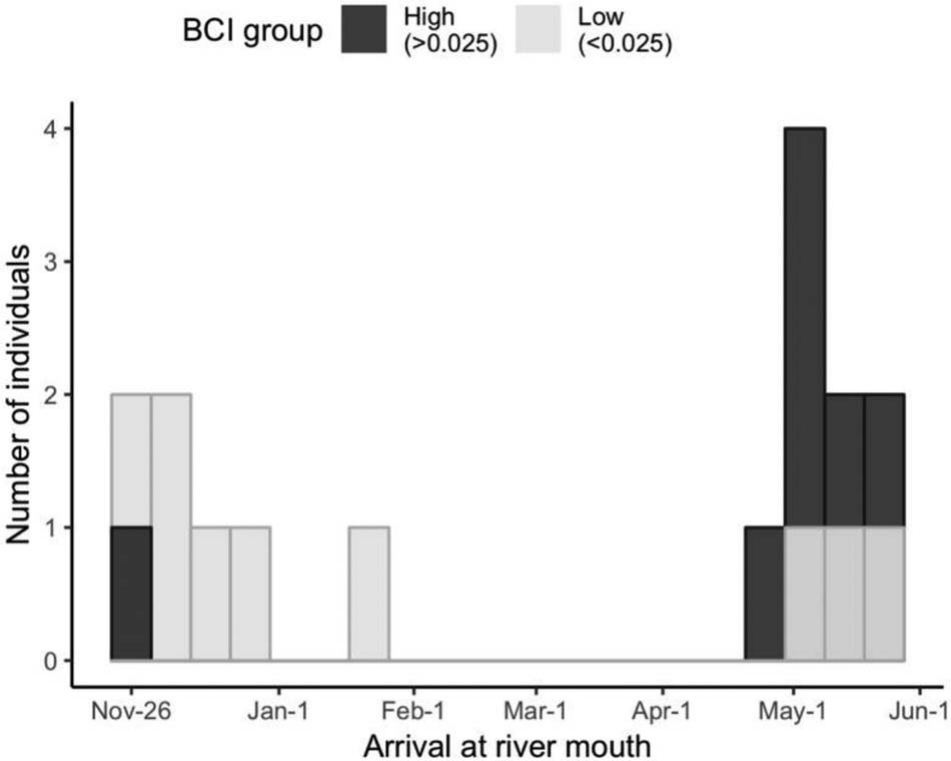
Histogram of the bimodal timing of arrival at the river mouth, with individuals in the high body condition group (>0.025) in black and low body condition group (<0.025) in grey (as previously defined in the Results section).

In support of our first prediction, the best-fitting (i.e. lowest AIC) binomial logistic regression model strictly included BCI (i.e. residual mass, Table 1), with kelts in lower condition having a higher probability of migrating soon after spawning (i.e. in late-fall or early-winter, Fig. 2) and spending the winter in the lower portion of the river. In contrast, kelts in higher body condition initiated downstream migration later in the spring. With a 0.1 increase in BCI (representing a mass gain of 313 g for a fish of 719 mm in fork length, for a total mass of 3294 g), the odds of waiting for the spring to initiate downstream migration as opposed to migrating down in the autumn were 2.6 times greater, for a change in probability from 56 (at BCI = −0.025) to 76% (at BCI = 0.075) (Fig. 2). BCI was not a statistically significant single predictor of the probability of autumn vs. spring downstream migration timing (binomial logistic regression, *P* = 0.104, Fig. 2). However, dividing kelts in two equal groups based on body condition scores, kelts in lower body condition (<0.025) migrated down to the river mouth earlier than kelts in higher body condition (>0.025) with an average group difference of 84 days (i.e. January 28 vs. April 21) (Fig. 3). Considering body condition as a categorical variable (i.e. low or high body condition), body condition was a statistically significant single predictor of the probability of autumn vs*.* spring downstream migration timing (binomial logistic regression, *P* = 0.016, Table 2). This formulation provided the most parsimonious model (i.e. lowest AIC, Table 1). Amongst the successful downstream migrants (*n* = 20), 90% (9/10) of kelts in the high body condition group spent the winter up-river and initiated their downstream migration in the spring, whereas only 30% (3/10) of kelts in the low body condition group did so, with the majority of these fish (70%) migrating down to the river mouth in the few weeks or months after spawning. The other explanatory variables, covariates and simple interactions considered (i.e. plasma triglycerides, sea-age at maturity, length and sex) were not retained in the model during the stepwise process (Table 1).

**Table 1:** Model selection process and composition of the best three fitting regression models (i.e. lowest AIC) for each migratory components

Rank	Model structure	AIC	ΔAIC	AICc
(i) Downstream migration timing (*binomial logistic*)				
1	~ BCI (cat.)	22.7	0.0	23.4
2	~ BCI	27.9	5.3	28.6
3	~ 1	28.9	6.2	29.1
(ii) Overwinter riverine survival *(binomial logistic)*				
1	~ SW + TRIG	26.4	0.0	27.6
2	~ SW	29.9	3.5	30.4
3	~ SW + BCI (cat.)	30.2	3.8	31.3
(iii) Estuarine residency period *(general linear)*				
1	~ BCI	133.4	0.0	135.2
2	~ BCI (cat.)	135.0	1.6	136.9
3	~ 1	135.6	2.2	136.5
(iv) Marine progression and overall survival *(Cox proportional hazards)*				
1	~ SW + TRIG + SW:TRIG	114.4	0.0	115.6
2	~ SW + TRIG	115.2	0.8	115.7
3	~ 1	117.6	3.2	NA

AICc is also presented for additional support. BCI (i.e. residual mass) as either a continuous or categorical variable (i.e. ‘cat.’ for low and high body condition group as detailed above). SW stands for sea-age at maturity (i.e. 1SW or 2SW) and TRIG for plasma triglyceride concentration.

**Table 2:** Composition of the best-fitting regression models (i.e. lowest AIC) for each migratory components

Variable	Coefficient	SE	*P*-value
(i) Downstream migration timing *(binomial logistic)*			
Intercept	2.20	1.05	—
BCI (cat. = low)	−3.05	1.26	0.016*
(ii) Overwinter riverine survival *(binomial logistic)*			
Intercept	−2.49	1.45	—
TRIG	4.58	2.31	0.047*
SW (1SW)	21.08	---	0.999
(iii) Estuarine residency period *(general linear)*			
Intercept	29.13	2.65	—
BCI	−61.54	29.81	0.057
(iv) Marine progression and overall survival *(Cox proportional hazards)*			
TRIG	−2.52	1.01	0.013*
SW (1SW)	−2.14	0.88	0.015*
TRIG:SW (1SW)	2.83	1.54	0.067

BCI (i.e. residual mass) as either a continuous or categorical variable (i.e. ‘cat’ for low and high body condition group as detailed above). SW stands for sea-age at maturity (i.e. 1SW or 2SW) and TRIG for plasma triglyceride concentration.

^*^ *P* < 0.05; *P* < 0.07.

### Overwinter riverine survival

None of the tagged kelts were detected on estuarine receivers of the Bras d’Or Lakes or on marine gates before spring, meaning that they all overwintered in the Middle River or near its mouth before migrating out to enter the estuary between 23 April and 25 May. Of the 25 individuals that were tagged, five kelts were never recorded at the river mouth (i.e. presumed to have died up-river). Another three individuals that were detected arriving at the river mouth in the autumn, and detected there for a minimum of ~2 months, were then never detected elsewhere in the array and were presumed to have died in the lower part of the river. Thus, the combined riverine overwinter survival of kelts was estimated at 68.0% (17/25).

**Figure 4:**
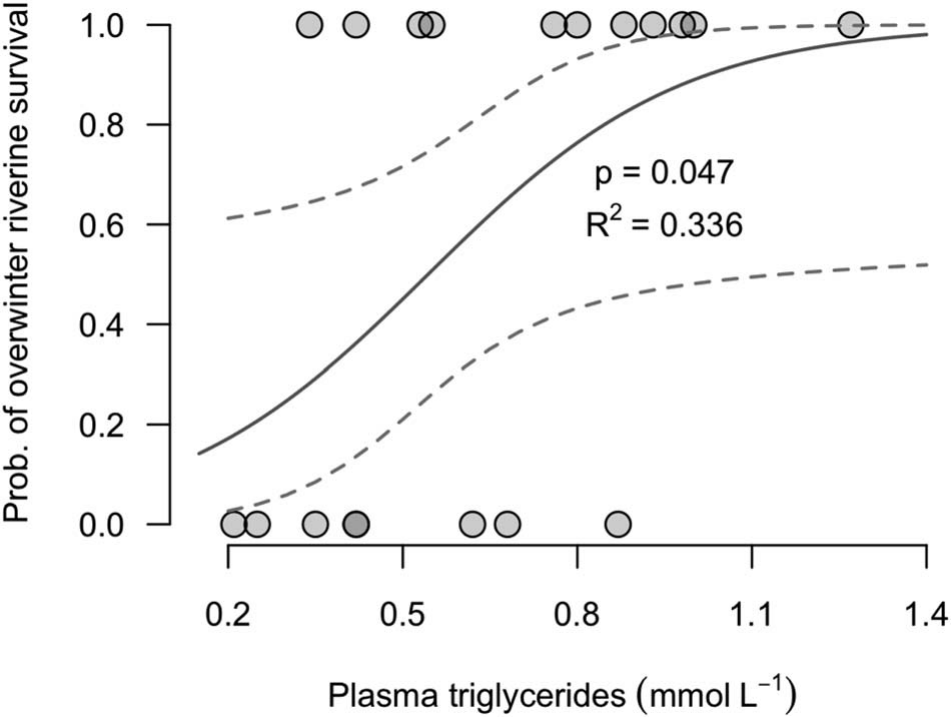
Binomial logistic regression of the probability of riverine overwinter survival as a function of plasma triglyceride concentration (only 2SW kelts are displayed as survival was perfect for 1SW kelts and not influenced by triglyceride level, Table 2). The coefficient of determination (*R*^2^) of the logistic regressions was computed using the *lrm* function of the *rms* package in R (Nagelkerke, 1991). Dashed lines represent the 95% CI.

In support of our second prediction, the best-fitting (i.e. lowest AIC score) binomial logistic regression model included plasma triglyceride level and sea-age at maturity as important predictors of riverine overwinter survival probability (Table 1), with depleted 2SW kelts dying to a greater extent. Whilst all 1SW kelts survived (6/6), compared to only 58% (11/19) for 2SW kelts, the odds of overwinter survival for 2SW kelts increased by 9.9 times with a 0.50 mmol l^−1^ increase in plasma triglycerides concentration (*P* = 0.047, Table 2 and Fig. 4). At the time of tagging the average concentration of plasma triglycerides for 2SW kelts was 1.6-fold higher in kelts that survived overwinter (0.77 mmol l^−1^) than in those that died in the few months after spawning (0.48 mmol l^−1^) (*P* = 0.024, Welch Two Sample *t*-test). The other explanatory variables, covariates (i.e. body condition, length and sex) and simple interactions considered were not retained in the model during the stepwise process. Because 1SW kelts were dominated by males (five males, one female) and 2SW kelts by females (one male, 18 females), the riverine overwinter survival probability of males (83% or 5/6) was higher than for females (63% or 12/19). However, we could not differentiate the effect of sex from the effect of sea-age at maturity due to strong correlation between these and relatively low sample sizes. The overwinter riverine survival of 1SW females (1/1) was equal to 1SW males (5/5). In contrast, the overwinter survival of 2SW females (11/18) appeared higher than that of 2SW males (0/1), although this remains uncertain due to low sample sizes.

### Estuarine survival and residency

All survivors that overwintered in the river (*n* = 17) entered the vast estuary formed by the Bras d’Or Lakes between 23 April and 25 May (median: 14 May). Amongst the individuals that were detected entering the estuarine habitat of the Bras d’Or Lakes (i.e. as detected on the Nyanza Bay gate), all were subsequently detected on the next two estuarine receiver gates leaving the estuary and entering the Atlantic Ocean between 9 May and 23 June (median: 12 June). Thus, the estuarine survival of kelts was 100% (17/17). Bras d’Or or estuarine residency periods varied amongst individuals between 5.9 and 45.1 days (average: 28.8 days).

**Figure 5:**
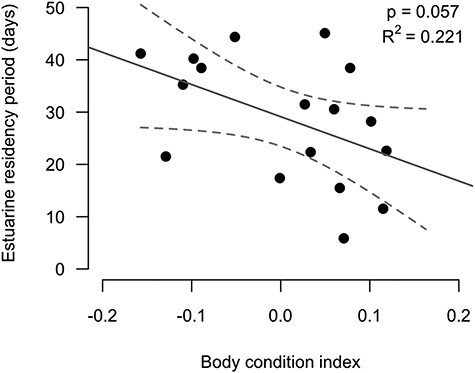
General logistic regression of the estuarine residency periods of kelts (*n* = 17) as a function of BCI (i.e. residual mass) (best-fitting model, Table 2). The Pearson correlation coefficient, *r* = −0.47. Dashed lines represent the 95% CI.

In partial support of our third prediction, the best-fitting (i.e. lowest AIC score) general linear regression model only included BCI (Table 1), indicating that depleted individuals tended to spend more time in the estuarine habitat of the Bras d’Or Lakes before migrating out to sea (6.2 extra days for each 0.1 decrease in BCI, and Pearson correlation coefficient, *r* = −0.47: Table 2 and Fig. 5). However, despite being the most parsimonious model (i.e. lowest AIC), BCI was only a marginally significant single predictor of the estuarine residency period of seaward migrating kelts (*P* = 0.057, Table 2). The other explanatory variables, covariates and simple interactions considered (i.e. plasma triglyceride, sea-age at maturity, length, and sex) were not retained in the model during the stepwise process (Table 1).

### Marine progression and overall survival pattern

Of the 17 kelts that were detected leaving the Bras d’Or Lakes to enter the Atlantic Ocean between 9 May and 23 June (median: 12 June), 13 were subsequently detected on the Cabot Strait receiver line between 16 May and 29 June (median: 15 June), providing a minimum early marine survival estimate of 76%. Six of these kelts were subsequently detected on the Strait of Belle Isle receiver array (located ~650 km from Middle River) between 3 July and 15 July (median: 11 July), presumably migrating towards the Labrador Sea. Of the initial 25 kelts that were tagged after spawning in late fall 2015, two kelts returned as repeat spawners. Both of these fish were female, 2SW-maiden kelts at tagging, and were tracked to the Strait of Belle Isle before coming back as alternate repeat spawners to the Middle River on 7 September and 10 September 2017, 489 and 504 days after having left the river in spring 2016. Thus, survival to repeat spawning was of 8% (2/25) for both sexes combined and 11% for females alone (2/19).

**Figure 6:**
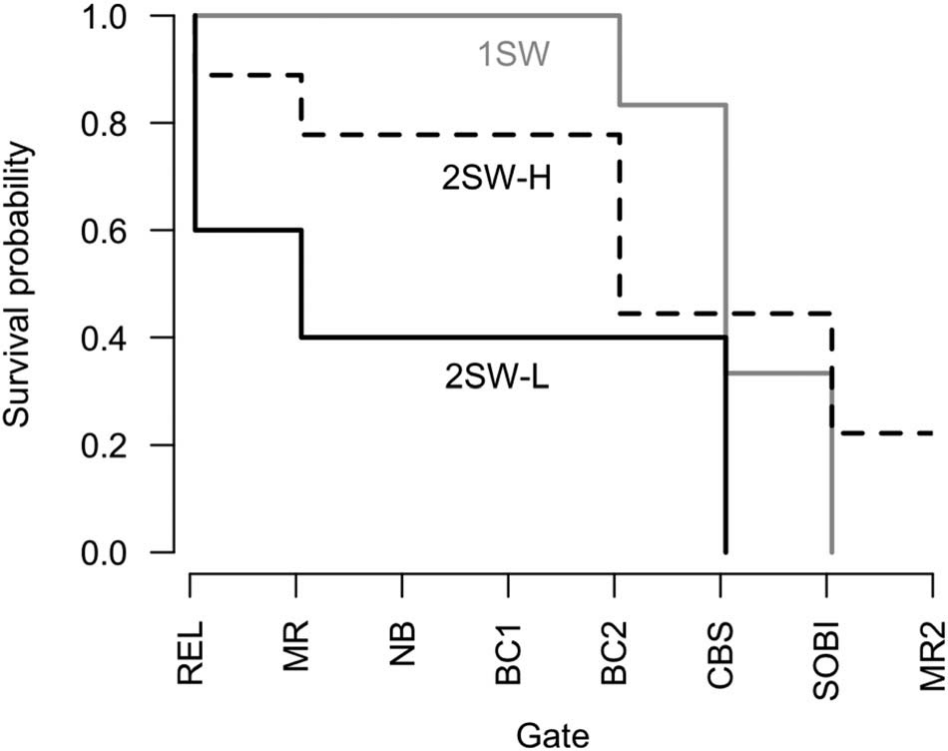
Kaplan-Meier product-limit survival fit of the progression or survival probability of kelts along the different stages of migration (from detections on different acoustic receiver gates) for three different groups based on sea-age at maturity and plasma triglyceride concentration (according to the best-fitting model, Table 2): all 1SW kelts (*n* = 6, solid pale grey line); 2SW kelts with high triglyceride level or 2SW-H (*n* = 9, dashed black line); and 2SW kelts with low triglyceride level or 2SW-L (*n* = 10, solid black line). REL is release site, MR is Middle River mouth, NB is Nyanza Bay, BC1 is the first gate of either Bras d’Or channels, BC2 is the second gate of either Bras d’Or channels, CBS is Cabot Strait, SOBI is Strait of Belle Isle, and MR2 is return to the Middle River mouth to repeat spawning (Fig. 1).

In support of our final prediction that the marine progression and overall survival probability would be reduced for nutritionally depleted individuals, the best-fitting (i.e. lowest AIC score) Cox proportional hazards regression model included plasma triglycerides level, sea-age at maturity and a simple interaction term between the two (Table 1). As such, the probability of progressing or surviving further through the different stages of migration (river, estuary, ocean, then back to the river to spawn again) increased with increasing plasma triglycerides level for 2SW kelts (*P* = 0.013, Table 2). And whilst post-spawning plasma triglycerides level was not a significant predictor of survival probability for 1SW kelts (considering the marginally significant interaction, *P* = 0.067, Table 2), 1SW kelts progressed on average further through the different stages of migration than 2SW kelts, as driven by their higher survival in earlier stages (Fig. 6). However, none of the 1SW kelts survived to spawn again. The other explanatory variables, covariates and simple interactions considered (i.e. body condition, length and sex) were not retained in the model during the stepwise process.

Based on these findings on the link between the overall survival of 2SW kelts and triglyceride level and to facilitate the interpretation, we divided 2SW kelts into two groups based on plasma triglycerides concentration: kelts with low post-spawning levels between 0.21 and 0.62 mmol l^−1^; and kelts with high levels between 0.68 and 1.27 mmol l^−1^. We then plotted Kaplan–Meier survival curves to better visualize differences in the progression or survival probability of kelts throughout the different stages of migration amongst 1SW (*n* = 6), 2SW with high triglyceride level (*n* = 9) and 2SW kelts with low triglyceride level (*n* = 10) (Fig. 6). 1SW kelts (independently of their plasma triglyceride concentration) had the highest survival probability through the riverine and estuarine migration stages with 100% of them surviving to reach the ocean (i.e. to BC2 gate, Fig. 1), followed by 2SW kelts with high triglyceride level at 78% survival, and by 2SW kelts with depleted triglyceride level at a reduced 40% survival probability (Fig. 6).

Once in the marine environment, the vast majority of 1SW kelts (5/6) and depleted 2SW kelts (4/4) that survived to enter the ocean, subsequently survived to cross the Cabot Strait. Whilst the early marine survival of both of these groups was high, the probability of progressing further (i.e. to cross the Strait of Belle Isle and enter the Labrador Sea) declined abruptly, with a reduced apparent overall survival of 33% (2/6) for 1SW kelts and 0% (0/10) for low triglyceride 2SW kelts (13% combined (2/16)) (Fig. 6). Moreover, none of these individuals returned to the Middle River to spawn again. In contrast, 44% (4/9) of 2SW kelts with high plasma triglyceride level survived to reach the Labrador Sea, with 22% (2/9) of them returning to the Middle River as alternate repeat spawners. These two individuals were 2SW females that had higher (i.e. 1.7-fold) post-spawning plasma triglyceride concentration than 2SW kelts that did not survive to spawn again (averages of 1.01 vs. 0.60 mmol l^−1^, respectively).

## Discussion

Our findings support the hypothesis that inter-individual differences in post-spawning nutritional condition underlie differential migratory decisions and survival propensity for Atlantic salmon kelts. We identified nutritional correlates of spatiotemporal variation in the habitat use and survival of kelts through their winter in river, during their seaward migration, and on their way back to spawn in subsequent years. Overall, kelts in depleted nutritional condition (i.e. low BCI or plasma triglyceride level): (i) initiated downstream river migration sooner; (ii) experienced higher overwinter riverine mortality; (iii) tended to spend greater time in the estuary before moving back to sea and (iv) did not progress as far in the marine environment and had a reduced probability of surviving to reproduce again than kelts in better nutritional condition.

After spawning, kelts in poor body condition had an increased probability of initiating downstream migration in the autumn to overwinter in the lower section of the river compared to animals with better body condition, which mostly overwintered up-river and only migrated down in the spring. Similar condition-dependent migration timing of Atlantic salmon kelts, with depleted individuals initiating downstream movement soon after spawning, was previously described by Halttunen *et al.* (2013). In addition to a lower body condition factor in autumn migrating Atlantic salmon kelts (Halttunen *et al.,* 2013), Birnie-Gauvin *et al.* (2019) reported that early migrants tended to have higher baseline plasma cortisol levels compared to spring migrating kelts. This might suggest that kelts perceive low nutritional state and low resource availability as a stressful stimuli, which might trigger the upregulation of cortisol levels for feeding and preparation for salt water. Although the majority of kelts with low body condition initiated downstream migration in the autumn, in contrast with previous studies (i.e. Halttunen *et al.,* 2013; Birnie-Gauvin *et al.,* 2019), no kelts from our study entered the sea before spring and instead decided to overwinter in the lower section of the Middle River or near its mouth. Complementary to our findings on the condition-dependent downstream migration timing of kelts, nutritional status was also linked to overwinter riverine survival, so that the survival probability of 2SW kelts decreased with decreasing post-spawning triglyceride concentration (i.e. an important source of energy for metabolic maintenance during starvation; Sargent *et al.,* 2002). In addition, all 1SW kelts survived through the winter months as expected given their lower metabolic costs due to proportionally lower reproductive investment (Jonsson *et al.,* 1991, 1997; Fleming, 1996). Amongst the kelts that successfully migrated down to the river mouth (either in the autumn or the spring) the only individuals that did not survive to reach the first estuarine gate were three 2SW females that had migrated down in the autumn. We believe this migratory decision about overwinter habitat choice reflects a trade-off between the safety of overwintering in freshwater and the more numerous feeding opportunities of the lower river section and adjacent estuarine habitat incurring greater risks, such as predation (as suggested by Halttunen *et al.,* 2013). Whilst no wild-spawned kelts in our study exited the river before spring, the risk of premature seaward migration was further exemplified in a recent study in the same system where the survival probability of wild-origin, hatchery-spawned kelts (i.e. high stress group) released back to the river after being stripped of gametes and which migrated to the estuary in the autumn was only 33% (Bordeleau *et al.,* 2018b). Together these findings suggest that depleted kelts are less likely to survive through the winter, likely due to a combination of higher nutritional requirements to sustain basal metabolic processes and the necessity to migrate to a risker habitat in trying to offset these. The overwinter riverine survival probability of kelts reported in our study (i.e. 68%) was of similar magnitude to that reported from the Alta River, northern Norway (63%) (Halttunen *et al.,* 2013).

In addition to the apparent role of nutritional condition in mediating the post-spawning downstream migration timing and overwinter riverine survival of Atlantic salmon kelts, we also identified nutritional correlates of spatiotemporal variation in the habitat use and survival of kelts through the estuarine and marine migration stages. As such, the estuarine residency period of kelts (of up to 45 days) was negatively correlated with body condition factor such that depleted kelts tended to spend more time in the brackish Bras d’Or Lakes before migrating to sea. With important numbers of rainbow smelt, *Osmerus mordax*, a preferred prey species for Atlantic salmon kelts (Cunjak *et al.,* 1998) present in the Middle River and the Bras d’Or Lakes in the winter and spring (Denny, unpublished work), we believe this provides the opportunity for an initial period of feeding needed to partially restore somatic reserves prior to oceanic migration (as suggested by Hedger *et al.,* 2009). Differential nutritional needs might explain the longer estuarine residency periods of kelts in lower body condition. Similarly, the marine residency period of adult anadromous brown trout was also negatively correlated with pre-migratory nutritional condition (i.e. plasma triglycerides, expressed at similar concentration to those in our current study) with depleted individuals spending more time feeding at sea, potentially reflecting higher energetic requirements (Bordeleau *et al.,* 2018a). Although no Atlantic salmon kelts that we tagged from the Middle River were detected in the brackish inland sea formed by the Bras d’Or Lakes after the month of June, the estuarine residency periods observed were substantial (i.e. up to 45 days in wild-spawned kelts; and even to 191 days in early migrating hatchery-spawned kelts: Bordeleau *et al.,* 2018b). This points out the ecological importance of this unique system for reconditioning Atlantic salmon, as suggested by Mikmaq traditional ecological knowledge (see Crossin *et al.,* 2016).

In our study, the survival of kelts through the estuary and the early marine migration phase (i.e. to cross the Cabot Strait and enter the Gulf of St. Lawrence) was 76%, which agrees with previous findings that these phases of the migration of Atlantic salmon kelts are characterized by high survival (range: 70–92%: Hubley *et al.,* 2008; Halttunen *et al.,* 2009; Hedger *et al.,* 2009). Moreover, whilst the early marine survival of kelts was high, the probability of them reaching the Labrador Sea declined abruptly to 35% (i.e. similar to that observed by Hedger *et al.,* 2009 at 32%). This decline in the progression probability of kelts in the later phase of their marine migration was especially important for 1SW and 2SW kelts with depleted triglyceride levels, compared to 2SW kelts with high triglyceride levels. Whilst this also seems to point out to the role of post-spawning nutritional condition in mediating longer-term migratory behaviour and survival of 2SW kelts, this is not evident for 1SW kelts that also experienced reduced progression probability in the marine environment. However, this might not solely reflect differences in survival as it might also have been partly driven by smaller (e.g. 1SW) and depleted kelts more likely to adopt a shorter distance migratory strategy to feed in the Gulf of St. Lawrence instead of migrating towards the Labrador Sea and the West coast of Greenland (Chaput and Benoit, 2012; Strøm *et al.,* 2017). In conjunction, it is also possible that smaller (e.g. 1SW) and depleted kelts experienced higher predation risks, which might be especially important in the Gulf of St. Lawrence (Strøm *et al.,* 2019). Nonetheless, it is perhaps unsurprising to note that amongst 2SW kelts the only two individuals that survived to repeat spawning were two female kelts with high post-spawning triglyceride levels. These two individuals were tracked all the way up to the Strait of Belle Isle before coming back to spawn as alternate repeat-spawners a few years after tagging.

Whilst we could not directly quantify total reproductive investment in our study, our findings are consistent with expectations from the life history trade-off between current reproduction and survival in which larger and more depleted individuals experienced higher post-spawning mortality as a result of higher energy investment into spawning (Jonsson *et al.,* 1991, 1997; Fleming, 1996; Aykanat *et al.,* 2019). This offers additional support for the biological relevance of measuring post-spawning plasma triglyceride concentration as a valuable and relatively simple non-lethal indicator of nutritional condition, and indicator of a potential reproductive carryover effect on post-spawn survival (as suggested by Bordeleau *et al.,* 2018a). Whilst additional research is needed to further clarify the link between total reproductive investment and post-spawning metabolites level, this might present future research opportunities in study designs, such as ours, were repeated sampling is not possible.

Collectively our findings highlight the role of nutritional condition in mediating the post-spawning migratory behaviour and survival of Atlantic salmon kelts. As such, indices of nutritional condition were correlated with spatiotemporal variation in the habitat use and survival of kelts throughout their entire migration (overwinter period, seaward migration and eventual return as repeat-spawners). This suggests that initial differences in post-spawning condition, likely determined by individual variation in reproductive investment, can be carried throughout their migration and ultimately affecting repeat-spawning potential. Moreover, by providing valuable information on some of the endogenous mechanisms at play, our findings will inform current and future management and conservation efforts directed at improving the survival prospects and future breeding potential of post-spawners. This is especially important considering recent findings on the ecological importance of repeat spawners to the viability of Atlantic salmon populations and the broad-scale spatiotemporal shifts in iteroparity that have been occurring over the last few decades across populations of the northwest Atlantic (Bordeleau *et al.*, 2019).
